# DNA Barcode-Based PCR-RFLP and Diagnostic PCR for Authentication of Jinqian Baihua She (Bungarus Parvus)

**DOI:** 10.1155/2015/402820

**Published:** 2015-05-19

**Authors:** Xiaolei Li, Weiping Zeng, Jing Liao, Zhenbiao Liang, Shuhua Huang, Zhi Chao

**Affiliations:** ^1^School of Traditional Chinese Medicine, Southern Medical University, Guangzhou 510515, China; ^2^Beijing Royal Intergrative Medicine Hospital, Beijing 102209, China; ^3^Department of Pharmacy, Zhongshan People's Hospital, Zhongshan 528403, China

## Abstract

We established polymerase chain reaction-restriction fragment length polymorphism (PCR-RFLP) and diagnostic PCR based on cytochrome C oxidase subunit I (COI) barcodes of *Bungarus multicinctus*, genuine Jinqian Baihua She (JBS), and adulterant snake species. The PCR-RFLP system utilizes the specific restriction sites of *Spe*I and *Bst*EII in the COI sequence of *B. multicinctus* to allow its cleavage into 3 fragments (120 bp, 230 bp, and 340 bp); the COI sequences of the adulterants do not contain these restriction sites and therefore remained intact after digestion with *Spe*I and *Bst*EII (except for that of *Zaocys dhumnades*, which could be cleaved into a 120 bp and a 570 bp fragment). For diagnostic PCR, a pair of species-specific primers (COI37 and COI337) was designed to amplify a specific 300 bp amplicon from the genomic DNA of *B. multicinctus*; no such amplicons were found in other allied species. We tested the two methods using 11 commercial JBS samples, and the results demonstrated that barcode-based PCR-RFLP and diagnostic PCR both allowed effective and accurate authentication of JBS.

## 1. Introduction

Jinqian Baihua She (JBS) (coin-like white-banded snake, Bungarus Parvus) is a widely used high-value traditional Chinese drug recorded in Chinese Pharmacopoeia (ChP, Vol.1, 2010 Edition). It derives from the dried body of infant* Bungarus multicinctus* Blyth and is effective in dispelling the wind, removing obstruction of the collaterals, and relieving spasm [1]. As attempts of domestication and breeding of* B. multicinctus* remain unsuccessful so far, the supply of JBS has to rely entirely on the gradually diminishing wild resource. The shortage in JBS supply results in its high price in market and also in the emergence of its adulterants using other snake species such as baby* Dinodon rufozonatum* and* B. fasciatus*. By now nine snake species have been found for sale under the name of JBS [2, 3], and according to our previous study, the adulterants accounted for about 50% of JBS crude drug market [4]. The conventional approach to JBS authentication depends solely on the macroscopic characters, which can be rather confusing even for experienced professionals [5, 6]. A convenient and accurate means for distinguishing genuine JBS from its adulterants is therefore urgently needed to ensure the safety and effectiveness of the drug use.

Recent developments in biological techniques and molecular genetic markers make JBS identification possible at the molecular level using species-specific DNA sequences. We previously demonstrated the efficiency of cytochrome C oxidase subunit I (COI) barcodes for identification of JBS against its adulterants [4]. However, sequence splicing and analysis involved in DNA barcoding requires knowledge in specific specialties, and sequence identification based on BLAST might result in hit of several species with a high sequence homology; in such cases, choosing an appropriate threshold for a decision becomes difficult. In addition, because conventional DNA sequencing is time-consuming and expensive, DNA barcoding technique has only limited application in routine practice of drug control, especially in elementary institutions in remote or underdeveloped areas.

Diagnostic polymerase chain reaction (PCR) (also called highly specific PCR) and polymerase chain reaction-restriction fragment length polymorphism (PCR-RFLP) were recently recorded in ChP for identification of several traditional Chinese drugs. In diagnostic PCR, a pair of primers is designed based on a specific gene fragment of the target species to amplify a species-specific DNA segment. In PCR-RFLP, the restriction sites are identified in a specific gene fragment of the target species, and with appropriate restriction enzymes, the gene fragment only of the target species is cleaved into 2 or more segments. These two methods, relatively simple and time-saving, can yield reliable results and meet the needs in practice of drug quality control [7–13]. In 2010, ChP recorded, for the first time, protocols of diagnostic PCR identification of Zaocys and Agkistrodon (derived from the snake species* Zaocys dhumnades* and* Deinagkistrodon acutus*, resp.) [1] and PCR-RFLP identification of Fritillariae Cirrhosae Bulbus [14]. As for JBS, reports have been available describing Cytb sequence-based identification of * B. multicinctus* against some adulterant snake species using species-specific primers and diagnostic PCR [15–17].

Our previous work has proved the feasibility and efficiency of COI barcodes in JBS authentication [4]. In the present study, we further established diagnostic PCR and PCR-RFLP systems based on the barcode sequences for differentiation of JBS from its adulterants. This is the first report describing COI barcode-based diagnostic PCR and PCR-RFLP for crude drug identification. Together with DNA barcodes system we previously reported and macroscopic identification, they compose an integrated system of JBS authentication.

## 2. Materials and Methods

### 2.1. Materials

Fifteen specimens of 11 snake species (including* B. multicinctus* and its adulterants) were obtained from different locations in Guangdong Province, Jiangxi Province, Hunan Province, and Guangxi Zhuang Autonomous Region in China (Table 1). Vouchers were deposited in School of Traditional Chinese Medicine, Southern Medical University, and all the specimens were preserved in 75% ethanol. Eleven samples of JBS crude drug were purchased from the local drug stores or crude drug market in Guangzhou, Guangdong Province (Table 2). All the snake specimens and crude drug samples were identified by Dr. Zhang Liang, South China Institute of Endangered Animals.

### 2.2. DNA Extraction

Tissue samples were dissected from the dorsal muscle of the snake. The total DNA was extracted using TIANamp Genomic DNA kit (Tiangen Biotech Co., Ltd., Beijing) following the manufacturer's instructions, dissolved in 100 *μ*L TE buffer, and stored at −20°C.

### 2.3. COI Barcode Amplification

The universal primers LCO1490 (5′-GGTCAACAAATCATAAAGATATTGG-3′) and HCO2198 (5′-TAAACTTCAGGGTGACCAAAAAATCA-3′) [18] were used to amplify the COI barcode region. A pair of primers designed for Viperidae snakes, DK1-CO1 (5′-CAACTAACCACAAAGACATCGG-3′) and DK1-CO2 (5′-CTTCTGGGTGGCCGAAAAACA-3′) [19], was used when the universal primers failed to obtain the target amplicon.

PCR reactions were carried out with an Applied Biosystems 2720 Thermal Cycler (Applied Biosystems, Carlsbad CA, USA), in a total volume of 25 *μ*L containing 12.5 *μ*L of 2 × Taq PCR MasterMix (Tiangen), 1 *μ*L of each primer, 1 *μ*L of genomic DNA, and 9.5 *μ*L of ddH_2_O. Thermal cycling was performed with an initial denaturing at 93°C for 5 min and annealing at 55°C for 2 min, followed by 35 cycles of 93°C for 30 s, 55°C for 45 s, and 70°C for 45 s, with a final extension at 70°C for 5 min and chilling to 4°C. The PCR products (5 *μ*L) were subjected to 1.2% agarose gel electrophoresis and visualized with ethidium bromide (EB) staining under UV.

PCR products were purified using Universal DNA Purification Kit (Tiangen Biotech Co., Ltd., Beijing) following the manufacturer's instructions and were in a final DNA concentration of 100 ng/*μ*L~500 ng/*μ*L.

### 2.4. Restriction Analysis of the PCR Products

COI barcode sequences of 15 specimens representing 11 snake species obtained in our previous studies were downloaded from BOLD system and GenBank. Restriction mapping of the COI sequences was carried out using the Nebcutter V2.0 [20]. Based on the restriction maps,* Spe*I and* Bst*EII were selected as candidate restriction endonucleases for discrimination between* B. multicinctus* and other snakes. Digestions with* Spe*I and* Bst*EII (Takara) were performed in a total volume of 20 *μ*L containing 2 *μ*L PCR products, 0.8 *μ*L enzymes, 14.4 *μ*L ddH_2_O, and 2 *μ*L 10 × digestion buffer at 37°C for 1 h as recommended by the manufacturer. The DNAs were fractionated by 2.0% agarose gel electrophoresis and visualized by EB staining under UV.

### 2.5. Species-Specific Primer Design and Diagnostic PCR

Fifteen COI sequences of 11 snake species were aligned with ClustalX and analyzed with Mega5.0 software. A pair of species-specific primers for* B. multicinctus* was designed based on COI sequences with the software Primer-Premier 5.0 and synthesized by Invitrogen Biotechnology (Shanghai) Co., Ltd. The reaction systems of diagnostic PCR are the same as those described in Section 2.3 except for the primers. Thermal cycling was performed with an initial step at 94°C for 5 min and 65°C for 2 min, followed by 25 cycles of 94°C for 30 s, 65°C for 50 s, and 72°C for 30 s, with a final extension at 72°C for 5 min and chilling to 4°C. The DNAs were fractionated by 2% agarose gel electrophoresis and visualized by EB staining under UV.

### 2.6. Identification of JBS Samples

Eleven JBS crude drug samples purchased were authenticated with the established PCR-RFLP and diagnostic PCR systems following the protocols described above. These samples were also identified by DNA barcoding; that is, the COI barcode region of these 11 samples were sequenced and analyzed with the identification engine provided by BOLD; the retrieved results were used to validate the established methods.

## 3. Results

### 3.1. COI Region Amplification

**Figure 1:**
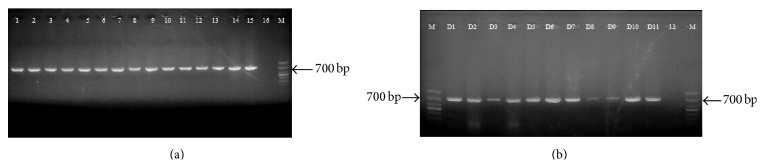
(a) Agarose gel electrophoresis of PCR results from BM1 (1), BM2 (2), BM3 (3), BM4 (4), BF (5), NJ (6), DR1 (7), DR2 (8), OM (9), EP (10), SA (11), DA (12), DF (13), ZY (14), XF (15), negative control (16) (water was used as sample), and DNA marker (M) in bp was indicated. (b) PCR products of the COI regions from JBS samples (D1–D11) and negative control (12) (water was used as sample) and DNA marker (M) in bp were indicated.

Using the primer pairs LCO1490 and HCO2198, or DK1-CO1 and DK1-CO2, a single PCR product about 700 bp was amplified (Figure 1). The amplification results of the DNA templates from the original animals and from snake crude drugs were identical.

### 3.2. Restriction Analysis

**Figure 2:**
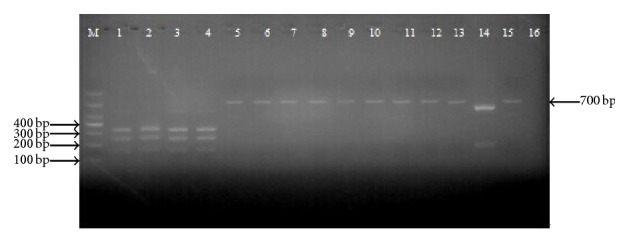
PCR-RFLP patterns of COI region digested with* Spe*I and* Bst*EII. BM1 (1), BM2 (2), BM3 (3), BM4 (4), BF (5), NJ (6), DR1 (7), DR2 (8), OM (9), EP (10), SA (11), DA (12), DF (13), ZY (14), XF (15), negative control (16) (water was used as sample), and DNA marker (M) in bp were indicated.

Analysis of the restriction maps revealed two restriction sites of endonucleases* Spe*I (A^▾^CTAGT) and* Bst*EII (G^▾^GTAACC) in the COI sequence of* B. multicinctus*, while that of* Z. dhumnades* had the restriction site of* Spe*I only; the other species contained neither* Spe*I nor* Bst*EII restriction site (Table 3). Thus, the COI region of* B. multicinctus* could be cleaved by* Spe*I and* Bst*EII into 3 fragments of 120 bp, 230 bp, and 340 bp. Because of the single* Spe*I restriction site in the COI region, the PCR product of* Z. dhumnades* was cleaved into only two fragments (120 bp and 570 bp) in the double endonuclease system. The PCR products of the other species could not be cleaved by the two endonucleases for the absence of restriction sites in the COI region (Figure 2).

### 3.3. Diagnostic PCR with Species-Specific Primers

**Figure 3:**
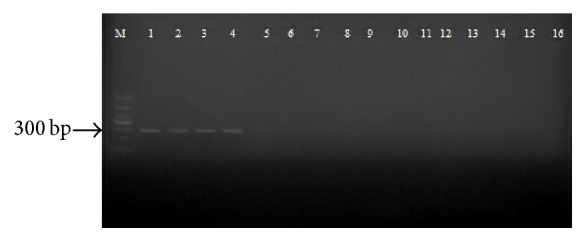
Diagnostic PCR for* B. multicinctus* and its adulterants. BM1 (1), BM2 (2), BM3 (3), BM4 (4), BF (5), NJ (6), DR1 (7), DR2 (8), OM (9), EP (10), SA (11), DA (12), DF (13), ZY (14), XF (15), negative control (16) (water was used as sample), and DNA marker (M) in bp were indicated.

The designed species-specific primers for identification of* B. multicinctus* COI37 (5′-AATCGGAGCCTGTCTAAG-3′) and COI337 (5′-GACTGTTCAACCTGTGCC-3′) are shown in Table 4. The forward and reverse primers are fully paired with the sequences of the COI region of* B. multicinctus*. The PCR conditions, especially the annealing temperature, were optimized. When the annealing temperature was raised to 65°C, only the template DNA of* B. multicinctus* could be amplified whereas the diagnostic PCRs yielded negative results for the other species. Under the optimized conditions, a single, distinct, and brightly resolved band of 300 bp was obtained only for* B. multicinctus*, while no amplification product was obtained for the others (Figure 3).

### 3.4. Commercial JBS Sample Identification

We tested 11 commercial JBS crude drug samples from the local market with the PCR-RFLP and diagnostic PCR. The universal primers LCO1490 and HCO2198, or the specifically designed primers DK1-CO1 and DK1-CO2, were used to amplify COI regions from the crude drug samples, and a single PCR product about 700 bp was observed (Figure 1).

**Figure 4:**
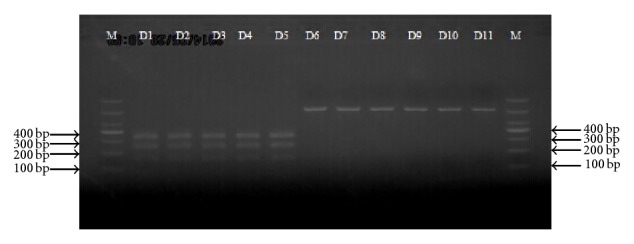
PCR-RFLP patterns of COI region from JBS samples (D1–D11) digested with* Spe*I and* Bst*EII, DNA marker (M) in bp were indicated.

PCR-RFLP analysis of the 11 commercial JBS samples was carried out using the double endonuclease system with* Spe*I and* Bst*EII. The PCR products (COI barcode region) of samples D1 to D5 could be digested into 3 fragments (120 bp, 230 bp, and 340 bp); these results were successfully repeated, indicating that these samples were authentic JBS. The COI fragments from the other 6 samples (D6–D11) could not be digested, indicating their identity as JBS adulterants (Figure 4).

**Figure 5:**
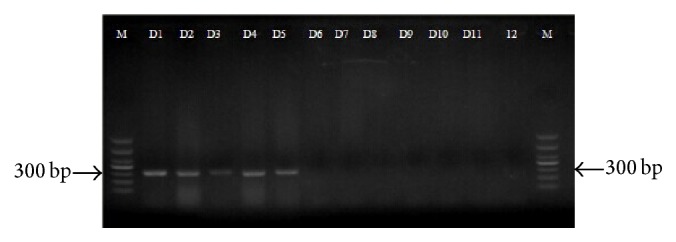
Diagnostic PCR products generated by primers (COI37 and COI337) using template DNA of JBS samples (D1–D11), negative control (12) (water was used as sample), and DNA marker (M) in bp were indicated.

Diagnostic PCRs of the 11 commercial JBS samples yielded a single, distinct, and bright band of 300 bp for samples D1–D5; no amplification product was obtained for the other samples. These results suggested that samples D1–D5 were from* B. multicinctus* (Figure 5).

Macroscopical inspection and DNA barcoding confirmed the results of PCR-RFLP and diagnostic PCR that samples 1–5 were derived from* B. multicinctus*. The identities of samples 6–11 determined by macroscopical inspection and DNA barcoding were listed in Table 2.

## 4. Discussion

*B. multicinctus*, the highly venomous many-banded krait, has long been used as a folk medicine in South China, especially in the tropical and subtropical mountainous regions around Nanling Mountains, that is, Guangdong, Guangxi, Hunan, Jiangxi, and so forth. Due to the hot summer and cold winter and high humidity almost all through the year, the residents in these areas often suffer from bone pain, arthritis, rheumatism, and even paralysis. To relieve or prevent these ailments, they preserved the snake in strong alcoholic drinks for months to allow the pharmaceutically active ingredients to be slowly released and regularly take the drinks. The effectiveness of such remedies has now been acknowledged in ChP, and the dried infant snakes (JBS) are thought to have stronger pharmacological actions.

However, artificial breeding of* B. multicinctus* remains unsuccessful, and JBS supply depends entirely on wild resources. The increasing demand of * B. multicinctus* in recent decades has drastically diminished their wild population and it is now listed in China Red Data Book of Endangered Animals [21] and IUCN Red List of Threatened Species 2012. As a result, the price of JBS increases considerably.

The high price of JBS gives rise to various versions of adulterants. The common practice of adulteration is decoloring the body bands of other snakes with decolorants, coloring the bands into white with paint, or even painting the bands on a snake without bands [22]. Such tricks were encountered in this study, as in JBS crude drug samples number 9 and number 11. Another practice faking JBS is splitting the body of large genuine snakes into small strips and assembling them with the heads of other snake species [22]. The differentiation of these adulterants from genuine JBS merely by their morphological characters is rather difficult because of their high resemblance in appearance, especially for the young snakes.

The development of molecular biological techniques provides a new solution for the identification of traditional Chinese medicinal materials [23, 24]. In 2010, PCR-RFLP and diagnostic PCR were officially adopted in ChP, and DNA barcoding would be included in the coming edition. Our previous studies indicated that COI barcodes had great advantages in the identification of JBS [4, 19, 25]. Compared with DNA barcoding technique, DNA barcode-based PCR-RFLP and diagnostic PCR are more convenient and efficient to distinguish JBS from its adulterants with reliable results; they can be complementary to DNA barcoding and macroscopical identification.

In PCR-RFLP, we initially considered single endonuclease digestion.* Spe*I, whose restriction site was located at bases 121 through 126 in the COI barcode region of* B. multicinctus*, allows restriction digestion of the sequence between bases 121 and 122. The barcode sequences of 9 reported JBS adulterants did not contain a* Spe*I restriction site, but when we expanded the investigation to more snake species, we found* Z. dhumnades* had the same sequences at bases 121 to 126 as* B. multicinctus*.* Bst*EII is another endonuclease specific to* B. multicinctus* and recognizes nucleotides at positions 353 to 359 to result in cleavage at position 353, yielding two fragments of 353 bp and 345 bp in length that can not be separated by electrophoresis. Single endonuclease digestion with either* Spe*I or* Bst*EII did not produce optimal results. But when combined, these 2 endonucleases cut COI amplicons of* B. multicinctus* into 3 fragments (340 bp, 230 bp, and 120 bp in length) and that of* Z. dhumnades* into 2 fragments (580 bp and 120 bp) without causing cleavage of the amplicons of other species (700 bp), thus allowing authentication of JBS crude drug samples. In addition, partial digestion did not occur with these 2 endonucleases as shown in previous studies [13].

Diagnostic PCR using primers specific to Cytb sequences had been applied in authenticating JBS [15–17]. In this study, for the first time, we successfully discriminated JBS from its adulterants with diagnostic PCR using species-specific primers based on COI barcode sequences. The primer pair COI37 and COI337 we used had a theoretical *T*_*m*_ value higher than 80°C to allow a high annealing temperature in PCR, which is an important factor for diagnostic PCR to improve the specificity. At the annealing temperature 55°C, the diagnostic PCR system amplified a DNA segment about 300 bp from the 4 template DNAs of* B. multicinctus*,* O. moellendorffi*,* Di. flavozonatum*, and* B. fasciatus*; at 60°C, the template DNA was amplified only from* B. multicinctus* and* B. fasciatus* of the same genus with only a 3-base difference in their primer sites. At an even higher annealing temperature of 65°C, the fragment was amplified only from* B. multicinctus* but not from other species or negative control. Under the optimized condition, the diagnostic PCR system could also distinguish* B. multicinctus* from other snake species such as* Ptyas mucosus*,* Lycodon ruhstrati*, and* Enhydris chinensis* (data not shown).

We tested 11 commercial JBS crude drugs with the established PCR-RFLP and diagnostic PCR systems. By both methods, samples D1–D5 were recognized as genuine JBS and samples D6–D11 as fake ones. Morphological identification and DNA barcodes analysis also indicated that samples D1–D5 were derived from* B. multicinctus*, whereas samples D6–D11 were not, suggesting the validity of these two methods in JBS authentication.

Molecular identification also has inherent limitations, such as its inability to determine whether the crude drug is derived from adult or juvenile snakes. Of the 11 commercial JBS samples we tested, sample D3 (Da Baihua She) was identified as genuine JBS of* B. multicinctus* origin by PCR-RFLP, diagnostic PCR, and DNA barcoding as well; but morphologically, it was a part of dried body of an adult* B. multicinctus* individual, which did not match the description of “dried body of infant snakes” in ChP. In such case, macroscopical inspection serves as a complementary means for molecular markers-based identification. On the other hand, molecular identification makes up for the inadequacy of identification based on morphological characters. The sample D11 could not be identified for origin through its appearance, but both PCR-RFLP and diagnostic PCR clearly suggested adulteration, and DNA barcoding further identified it as* Di. rufozonatum*.

The PCR-RFLP and diagnostic PCR systems we established, combined with macroscopical identification and DNA barcoding, constitute a comprehensive approach to JBS identification. These methods can be used independently or in different combinations according to the objective of the work, laboratory conditions, and the advantages and disadvantages of each method. For the purpose of authentication only without considering the zoological origin, either PCR-RFLP or diagnostic PCR will suffice because both of them are rapid, simple, cost-effective, and time-saving. Further investigation of the zoological origin of an adulterant requires the use of DNA barcoding for a conclusive result.

## 5. Conclusions

We for the first time established COI barcode-based PCR-RFLP and diagnostic PCR systems for effective authentication of JBS. These two methods can be integrated with macroscopical identification and DNA barcoding technique to constitute a comprehensive authentication and identification system for JBS.

## Figures and Tables

**Table 1:** Samples used in this study.

Sample	Species	Collecting site	GenBankID	BINs
BM1	*Bungarus multicinctus *	Zhongshan, Guangdong	JN833585	AAF9297
BM2	*B. multicinctus *	Taishan, Guangdong	JN833594	AAF9297
BM3	*B. multicinctus *	Conghua, Guangdong	JN833596	AAF9297
BM4	*B. multicinctus *	Maoming, Guangdong	JN860064	AAF9297
BF	*B. fasciatus *	Guangxi	JN833615	AAI8427
DA	*Deinagkistrodon acutus *	Jiangxi	JQ658431	ACC5654
DR1	*Dinodon rufozonatum *	Zhongshan, Guangdong	JN833598	AAD0172
DR2	*Di. rufozonatum *	Hunan	JN833601	AAD0172
DF	*Di. flavozonatum *	Shaoguan, Guangdong	JX233649	
OM	*Orthriophis moellendorffi* *(Elaphe moellendorffi) *	Guangxi	JN833617	ABA1479
EP	*Enhydris plumbea *	Zhongshan, Guangdong	JN833609	AAJ0753
NJ	*Naja atra *	Taishan, Guangdong	JN833602	AAF7608
SA	*Sinonatrix annularis *	Zhongshan, Guangdong	JN833613	ABA1332
XF	*Xenochrophis flavipunctatus *	Zhongshan, Guangdong	JN833606	AAH9231
ZY	*Zaocys dhumnades *	Conghua, Guangdong	JX233651	

**Table 2:** Identification results of JBS crude drug samples.

Samples	Commodity name	Producing area	Macroscopical inspection	PCR-RFLP	Diagnostic PCR	DNA barcoding	Sequence similarity
D1	Xiao Baihua She	Guangxi	*B. multicinctus *	+	+	*B. multicinctus *	100%
D2	Xiao Baihua She	Guangxi	*B. multicinctus *	+	+	*B. multicinctus *	100%
D3	Da Baihua She	Guangxi	*B. multicinctus *	+	+	*B. multicinctus *	100%
D4	Xiao Baihua She	Hunan	*B. multicinctus *	+	+	*B. multicinctus *	99.85%
D5	Jinqian Baihua She	Guangdong	*B. multicinctus *	+	+	*B. multicinctus *	100%
D6	Baihua She	Guangdong	*Di. rufozonatum *	−	−	*Di. rufozonatum *	99.85%
D7	Baihua She	Unknown	*Di. rufozonatum *	−	−	*Di. rufozonatum *	99.69%
D8	Baihua She	Unknown	*S. annularis *	−	−	*S. annularis *	99.24%
D9	Baihua She	Unknown	*En. chinensis *	−	−	*En. chinensis *	98%
D10	Baihua She	Unknown	*Di. rufozonatum *	−	−	*Di. rufozonatum *	99.54%
D11	Xiao Baihua She	Guangxi	Colubridae sp.	−	−	*Di. rufozonatum *	99.24%

+: Jinqian Baihua She; −: adulterant.

**Table 3:** Two restriction endonucleases selected for the identification of *B. multicinctus* based on COI sequences among eleven species.

	*Spe*I		*Bst*EII
	121		353
BM	A^*▾*^CTAGT **⋯ ⋯⋯⋯** G^*▾*^GTAACC		
DR	T CTAGT **⋯⋯⋯⋯** G GAAACC		
NJ	A CTTGT **⋯⋯⋯⋯** G GAAACC		
XF	A TTGAT **⋯⋯⋯⋯** G GTAACT		
EP	C CTAGT **⋯⋯⋯⋯** G GAAACC		
SA	C CTAGT **⋯⋯⋯⋯** G GAAACC		
BF	G TTAGT **⋯⋯⋯⋯** G GTAACT′		
OM	T CTGGT **⋯⋯⋯⋯** G GAAATC		
DA	C CTAGT **⋯⋯⋯⋯** G GAAACC		
ZY	A^*▾*^CTAGT **⋯⋯⋯⋯** G GAAACC		
DF	T CTAGT **⋯⋯⋯⋯** G GAAACC		

**Table 4:** Primer sites of species-specific primers COI37 and COI337 designed for the identification of *B. multicinctus* based on COI sequences among 11 species.

	37																	54
BM	A	A	T	C	G	G	A	G	C	C	T	G	T	C	T	A	A	G
DR	.	.	.	.	.	.	G	.	.	T	.	.	C	.	.	T	.	.
NJ	.	.	.	.	.	.	G	.	.	.	.	.	C	.	.	.	.	.
XF	.	.	.	T	.	.	.	.	.	T	.	.	C	.	.	.	.	.
EP	.	.	.	.	.	.	C	.	.	.	.	.	C	.	.	.	.	.
SA	.	.	.	.	.	.	.	.	.	.	.	.	C	.	.	.	.	.
BF	.	.	.	.	.	.	.	.	.	.	.	.	C	.	.	.	.	.
OM	.	.	.	T	.	.	G	.	.	T	A	.	.	.	.	.	.	.
DA	T	.	.	A	.	.	.	.	.	.	.	.	C	.	.	.	.	.
ZY	.	.	.	.	.	G	.	.	A	.	.	C	.	.	.	.	.	.
DF	T	.	.	.	.	.	.	.	.	T	.	.	C	.	.	T	.	.

	320																	337

BM	G	G	C	A	C	A	G	G	T	T	G	A	A	C	A	G	T	C
DR	.	.	.	.	.	.	.	.	G	.	.	.	.	.	A	.	.	.
NJ	.	.	T	.	.	C	.	C	.	.	.	.	.	.	.	.	.	.
XF	.	.	.	.	.	C	.	C	.	.	.	.	.	.	.	.	.	G
EP	.	.	.	.	.	.	.	A	.	.	.	.	.	.	.	.	.	A
SA	.	.	.	.	.	C	.	G	.	.	.	.	.	.	.	.	.	G
BF	.	.	.	.	.	.	.	C	.	.	.	.	.	.	.	.	.	T
OM	.	.	.	.	.	.	.	G	.	.	.	.	.	.	.	.	C	.
DA	.	.	A	.	.	.	.	.	.	.	.	.	.	.	C	.	.	.
ZY	.	.	.	.	.	T	.	G	.	.	.	.	.	.	.	.	.	A
DF	.	.	T	.	.	.	.	A	.	.	.	.	.	.	C	.	.	A
